# Perioperative anesthetic management in pediatric heart transplantation: a single-center descriptive study involving 27 patients

**DOI:** 10.3389/fped.2026.1842135

**Published:** 2026-06-18

**Authors:** Ya-fei Cheng, Cong-li Meng, Yi-ru Wang, Lin Chen, Ya-qun Ma, Wen-zhi Guo, Hang Guo

**Affiliations:** 1 Department of Anesthesiology, The Seventh Medical Center to Chinese PLA General Hospital, Beijing, China; 2 The Second School of Clinical Medicine, Southern Medical University, Guangzhou, China

**Keywords:** end-stage heart failure, extracorporeal membrane oxygenation, pediatric cardiac anesthesia, pediatric heart transplantation, perioperative anesthesia, perioperative complications

## Abstract

**Background:**

Orthotopic heart transplantation is the standard treatment for pediatric end-stage heart disease. Due to the wide age range and high physiological heterogeneity among pediatric recipients, perioperative anesthesia management is particularly complex. Literature describing real-world perioperative practices for high-risk populations remains limited.

**Objective:**

This descriptive study aims to summarize the perioperative anesthesia management strategies and short-term clinical outcomes for pediatric heart transplant recipients at a single center.

**Methods:**

This retrospective descriptive study enrolled 27 pediatric patients who underwent orthotopic heart transplantation at a single center from May 2022 to October 2025. Data on baseline characteristics, intraoperative variables, complications, and outcomes were collected. No control group was established, and no causal inference was performed.

**Results:**

he cohort included 27 patients (13 male, 14 female), aged 6 months to 16 years (weight 7.0–95.0 kg). Dilated cardiomyopathy was the most common diagnosis (20/27, 74.1%). All patients were NYHA class III–IV; median LVEF was 28.5%. Preoperative ECMO support was used in 15 patients (55.6%). No major cardiovascular events occurred during induction or pre-CPB. Mean CPB time was 183.3 ± 55.6 min; mean cold ischemia time was 249.5 ± 101.1 min. Mean postoperative mechanical ventilation was 7.7 ± 9.5 days. Postoperative ECMO was required in 11 patients (40.7%). At follow-up, 23 patients were alive and 4 died (survival 85.2%). Major complications included hypoxic-ischemic encephalopathy (*n* = 4), AKI requiring CRRT (*n* = 5), and peripheral nerve injury (*n* = 2).

**Conclusion:**

Among the pediatric heart transplant recipients with severe conditions in this cohort (including a high proportion of ECMO bridged cases), the perioperative anesthesia management strategy implemented included low-dose staged induction, invasive monitoring, and multidisciplinary collaboration. Pulmonary hypertension, primary graft dysfunction, and multi-organ complications were commonly observed in this cohort. This study is descriptive and observational; causal relationships cannot be inferred, and the findings require further validation in larger sample sizes or multicenter studies.

## 1 Introduction

In 1967, Christiaan Barnard successfully performed the first adult heart transplantation in Cape Town, South Africa, and later that same year attempted the first infant heart transplantation (1). However, the recipient died within hours due to severe metabolic and respiratory acidosis, highlighting the significant perioperative challenges associated with early pediatric heart transplantation (1). Over the past few decades, advancements in surgical techniques, immunosuppressive therapy, heart failure management, organ preservation, perioperative care, and multicenter collaborative research have markedly improved outcomes of heart transplantation. Consequently, both the number and survival rates of pediatric heart transplants have steadily increased (2). According to data from the International Society for Heart and Lung Transplantation (ISHLT), a total of 15,726 pediatric heart transplants had been performed globally as of June 30, 2018. The 1-year survival rate rose from 87% between 2000 and 2005 to 92% between 2012 and 2017, while the conditional 5-year survival rate increased from 88% between 2002 and 2007 to 90.5% between 2008 and 2013 (3). Orthotopic heart transplantation has thus become the standard treatment for infants and children with end-stage congenital heart disease or cardiomyopathy.

In China, pediatric heart transplantation started relatively late, with the first case performed in 1994, but has since developed rapidly. In 2024, China performed a total of 1,064 heart transplant surgeries, a 7% increase compared to 2023; among these, 148 cases (13.9%) involved pediatric patients under 18 years of age (3). Data from 2015 to 2024 show that the 30-day, 1-year, 3-year, and 5-year survival rates for pediatric heart transplant recipients in China were 94.7%, 87.6%, 74.5%, and 70.0%, respectively (3). Although progress has been made in pediatric heart transplantation in China in recent years, its development remains constrained by donor shortages, uneven regional distribution of medical resources, and the limited overall number of cases.

Currently, detailed descriptions specifically addressing perioperative anesthesia management in pediatric heart transplantation remain limited in the literature. Most available evidence derives from adult studies or small case reports. Due to significant differences between children and adults in cardiovascular physiology, drug responses, and complication profiles, findings from adult transplantation studies cannot be directly applied to pediatric patients (4). Furthermore, the wide age range and high physiological heterogeneity among pediatric heart transplant recipients make perioperative management more complex than in adults, particularly regarding anesthesia strategies, circulatory support, and multi-organ protection. Therefore, systematic descriptive reports on real-world perioperative practices for this population are urgently needed.

Therefore, in this study, our aim is to provide a detailed descriptive report on the perioperative anesthesia management and short-term clinical outcomes for pediatric heart transplant recipients in China. This article describes the strategies we employed, the observed complications, and the short-term outcomes of a cohort comprising a high proportion of critically ill patients (treated with extracorporeal membrane oxygenation bridging). Given the descriptive and retrospective nature of this study, no causal inferences are drawn, nor were control groups established. Our objective is to share practical insights that inform future hypotheses, aiming to serve as a reference for clinical practice and support the design of future multicenter studies.

## 2 Materials and methods

### 2.1 Patient population

This study is a single-center, retrospective, descriptive observational study. No control group was established, no causal hypothesis testing was conducted, and no predictive model was developed. All donor hearts were obtained through organ donation and allocated via the China Human Organ Allocation and Sharing Computer System. Data were retrospectively collected from 27 pediatric heart transplantation cases performed at the Seventh Medical Center of the People's Liberation Army General Hospital between May 2022 and October 2025. Preoperative baseline data, donor information, intraoperative parameters, postoperative complications, and follow-up data were extracted from medical records.

Inclusion criteria:

1. The patient must be under 18 years of age at the time of transplantation surgery;
2. First-time, single-organ, and orthotopic heart transplantation.

Exclusion Criteria:

1. Combined multi-organ transplantation;
2. Any history of previous organ transplantation;
3. Severe data gaps during the perioperative period (>30% of key variables cannot be traced).

### 2.2 Indications and contraindications for pediatric heart transplantation

Pediatric heart transplantation is generally indicated for end-stage cardiomyopathy, complex congenital heart disease not amenable to conventional surgical correction and associated with severe heart failure or hypoxemia, and other irreversible cardiac diseases in which symptoms cannot be improved despite palliative or corrective interventions. Contraindications include active malignancy, uncontrolled infection, and adverse psychosocial conditions that may limit the expected benefit of transplantation. Patients with severe irreversible dysfunction of other organs are generally not considered candidates for isolated heart transplantation and may require combined multiorgan transplantation. In addition, patients with severe, irreversible fixed pulmonary vascular resistance or marked hypoplasia of the central pulmonary arteries or pulmonary veins are generally not considered suitable candidates for heart transplantation (5).

### 2.3 Preoperative management

Many patients in this cohort had advanced heart failure and hemodynamic instability. Preoperative transport and anesthetic preparation in our center were directed at maintaining circulatory support and oxygenation. Preoperative sedatives were used cautiously. In this cohort, no cases of myocardial depression, reduced cardiac output, or worsening circulatory failure were observed in association with sedative administration. All ongoing therapies, including vasoactive agents, pulmonary vasodilators (e.g., treprostinil), and ECMO support, were maintained continuously during transport and anesthetic induction. Interruption of these therapies was avoided in this cohort. In patients requiring preoperative mechanical ventilation, adequate oxygenation was maintained during transport. Hypoxia-induced or worsening pulmonary hypertension was not observed in this cohort during transport. Before anesthetic induction, an experienced cardiac surgeon and a perfusionist were present and available for immediate intervention. For patients with an implanted left ventricular assist device (LVAD), a trained specialist remained on standby throughout the procedure until device explantation.

Standard intraoperative monitoring included five-lead electrocardiography (ECG), pulse oximetry (SpO_2_), end-tidal carbon dioxide (PetCO_2_), nasopharyngeal and rectal temperatures, bispectral index (BIS), and cerebral oxygen saturation. Invasive arterial pressure monitoring was established through radial or femoral arterial cannulation under ultrasound guidance. Central venous access was obtained through the internal jugular or subclavian vein for central venous pressure monitoring. In selected patients (for example, those with suspected pulmonary hypertension, right ventricular dysfunction, or hemodynamic uncertainty), a Swan-Ganz catheter was inserted through the right internal jugular vein to monitor pulmonary artery pressure.

Strict aseptic technique was used for all invasive procedures.Donor procurement and recipient surgery were closely coordinated. In our center, recipient anesthetic induction was initiated after visual confirmation of donor heart suitability by the procurement team.

### 2.4 Anesthetic induction and maintenance

Given the limited cardiac reserve of these patients, anesthetic induction was performed using a low-dose, slow, stepwise titration strategy to minimize cardiovascular depression and maintain hemodynamic stability.

In infants and young children, induction was typically achieved with midazolam (0.05–0.1 mg/kg), fentanyl (10–20 μg/kg), and rocuronium (0.6–1 mg/kg) in 5 patients, while agents with marked myocardial depressant effects, such as propofol, were avoided. Anesthesia was maintained with intermittent fentanyl and rocuronium boluses combined with inhaled sevoflurane. Given the anatomical characteristics of the pediatric airway, tracheal intubation was performed gently using appropriately sized cuffed endotracheal tubes to minimize trauma and air leakage. Correct tube placement was confirmed before mechanical ventilation. Ventilator settings were adjusted according to age, with a tidal volume of 6–8 mL/kg, respiratory rate of 20–30 breaths/min, and an inspiratory-to-expiratory ratio of 1:1.5 to 1:1, while excessive airway pressures were avoided.

In older children, induction commonly included midazolam (1–3 mg), etomidate (0.2–0.3 mg/kg), sufentanil (0.5–1 μg/kg), and rocuronium (0.6–1 mg/kg). Maintenance anesthesia consisted of remifentanil, dexmedetomidine, sevoflurane, and rocuronium. In school-aged children, appropriately sized endotracheal tubes were selected. Ventilator settings included a tidal volume of 8–10 mL/kg and a respiratory rate of 15–20 breaths/min, maintaining PetCO_2_ between 35 and 45 mmHg. Topical anesthesia, such as lidocaine spray, was used during intubation when necessary to reduce airway stimulation. Because orthotopic heart transplantation is a semi-emergent procedure owing to the unpredictability of donor availability, modified rapid-sequence induction was used in patients without adequate preoperative fasting. Gastric ultrasound was used when necessary to assess aspiration risk; a gastric fluid volume of less than 1.25 mL/kg was considered indicative of a low risk of reflux and aspiration (6).

### 2.5 Anesthetic management during cardiopulmonary bypass

After median sternotomy and surgical exposure, systemic heparinization was administered before CPB cannulation at a dose of 350–400 U/kg. In patients receiving preoperative anticoagulation, possible heparin resistance due to antithrombin III deficiency was considered, and additional heparin or antithrombin III concentrate was administered as needed (7). Activated clotting time (ACT) was monitored intraoperatively and maintained above 400 to 500 s. After initiation of CPB, the vena cavae and aorta were sequentially clamped, followed by systemic cooling and administration of histidine-tryptophan-ketoglutarate solution for myocardial protection and cardiac arrest. Mechanical ventilation was discontinued, and a continuous positive airway pressure of 5–10 cmH_2_O was maintained by adjusting the anesthesia circuit adjustable pressure-limiting valve, with a fraction of inspired oxygen (FiO_2_) of 0.4–0.6 and a gas flow rate of 0.5–1 L/min to keep the lungs inflated. Peripheral perfusion was closely monitored during CPB; warm extremities, capillary refill time of less than 2 s, and urine output of at least 1 mL/kg/h were considered indicators of adequate tissue perfusion. Surgical manipulation during cardiac dissection and cannulation was associated with arrhythmias in some cases. Anesthetic depth was carefully titrated in this cohort, and excessively light anesthesia was avoided. Additional opioids were administered before intense surgical stimulation. Tachycardia-induced myocardial ischemia and arrhythmias were not observed in this cohort following this practice. The BIS was maintained between 40 and 60 throughout the procedure.

### 2.6 Antibiotic prophylaxis and immunosuppressive therapy

Prophylactic antibiotics were administered before skin incision and included imipenem–cilastatin (15 mg/kg), either alone or in combination with vancomycin. Esomeprazole (0.5–1.5 mg/kg/day) was also administered for gastric acid suppression. At the time of pericardiotomy, basiliximab was administered intravenously in 23 patients at a dose of 20 mg for body weight less than 35 kg and 40 mg for body weight 35 kg or greater. In 4 patients, a recombinant humanized anti-CD25 monoclonal antibody was administered at 1 mg/kg within 24 h before surgery. Methylprednisolone (10 mg/kg; maximum, 500 mg) was administered before aortic unclamping during CPB. Postoperatively, rejection prophylaxis consisted of methylprednisolone sodium succinate, mycophenolate mofetil, and tacrolimus.

### 2.7 Post-CPB anesthetic and hemodynamic management

After donor heart implantation, all anastomoses were carefully inspected, and rewarming was initiated. Vasoactive agents were started before aortic unclamping, followed by sequential release of the aorta and vena cavae.

During reperfusion and the parallel circulation phase, continuous dopamine (3–15 μg/kg/min) and epinephrine (0.03–0.1 μg/kg/min) infusions were routinely administered. Norepinephrine (0.03–0.1 μg/kg/min) was added when hemodynamic stability was not maintained with the initial vasoactive regimen. In patients with right ventricular dysfunction, milrinone (0.375–0.75 μg/kg/min) or dobutamine (3–15 μg/kg/min) was administered. Heart rate support was provided with isoproterenol (0.03–0.1 μg/kg/min), and temporary pacing was used as needed, with pacing rates adjusted to the patient's age. Ventricular arrhythmias can occur during reperfusion or weaning from CPB (8). Management strategies included optimization of reperfusion, internal defibrillation (10–30 J), maintenance of serum potassium above 5 mmol/L, magnesium supplementation (10–20 mmol), and administration of antiarrhythmic agents such as amiodarone or lidocaine (1 mg/kg). In patients with pulmonary hypertension, inhaled nitric oxide and continuous treprostinil infusion (initial dose, 2 ng/kg/min) were administered and titrated according to pulmonary artery pressure to improve pulmonary hemodynamics without compromising systemic circulation. Pulmonary and systemic pressures were continuously monitored in patients with pulmonary artery catheters in place.

Weaning from CPB was attempted only after the following criteria were met: (1) nasopharyngeal temperature of 36–37°C and rectal temperature above 35°C; (2) stable arterial pressure, with systolic pressure of 80–100 mmHg during reduction of bypass flow; (3) adequate heart rate, defined as 80–100 beats/min in older children and 120–140 beats/min in infants; (4) no significant surgical bleeding; (5) hemoglobin of at least 80 g/L in older children and at least 90 g/L in infants; (6) stable blood gas and electrolyte values; and (7) absence of severe arrhythmias, with sinus rhythm achieved spontaneously or after pharmacologic treatment or pacing (9). Before protamine administration, arterial pressure was increased slightly. Heparin was reversed with slowly administered protamine at a 1:1.5 ratio under close monitoring of airway pressure, blood pressure, heart rate, and the surgical field to detect adverse reactions (10). In the event of a protamine reaction, protamine administration was immediately discontinued, and intravenous methylprednisolone (40 mg), increased inotropic support, bronchodilation, and diuresis were initiated to maintain hemodynamic stability. After low-dose protamine desensitization, heparin reversal was cautiously continued. If circulatory collapse occurred, CPB was promptly re-established. When moderate-to-high vasoactive support, defined as epinephrine or norepinephrine above 0.1–0.2 μg/kg/min, failed to maintain adequate circulation, veno-arterial extracorporeal membrane oxygenation (VA-ECMO) or temporary right ventricular assist support was considered.

### 2.8 Blood product preparation

Exposure to allogeneic blood products may induce the formation of human leukocyte antigen antibodies, which is associated with adverse outcomes after heart transplantation. In this cohort, allogeneic blood products were used. All blood products were leukocyte-depleted. Preoperative blood product preparation was tailored according to coagulation status, prior cardiac surgical history, and laboratory findings. Packed red blood cells, fresh frozen plasma, platelets, and fibrinogen concentrate were prepared in this cohort.

### 2.9 Statistical analysis

Only descriptive statistics were employed. Measurement data were expressed as mean ± standard deviation or median (range), while categorical data were presented as frequency and percentage (%). No intergroup comparisons, regression analyses, or causal inferences were conducted.

## 3 Results

### 3.1 Preoperative severity and baseline characteristics

Between May 2022 and October 2025, a total of 27 pediatric patients underwent orthotopic heart transplantation at our center and were included in this descriptive analysis. The cohort comprised 13 male and 14 female patients, with an age range of 6 months to 16 years and a weight range of 7.0 kg to 95.0 kg. Dilated cardiomyopathy was the most common primary diagnosis, occurring in 20/27 patients (74.1%). Other diagnoses included post-myocarditis cardiomyopathy (3/27, 11.1%), postoperative congenital heart disease (3/27, 11.1%), and restrictive cardiomyopathy (1/27, 3.7%). All 27 patients (100%) had a New York Heart Association (NYHA) functional classification of class III or IV before transplantation. The median left ventricular ejection fraction was 28.5% (range: 12%–64%). Fifteen patients (55.6%) received preoperative extracorporeal membrane oxygenation (ECMO) support.

### 3.2 Intraoperative management and perioperative variables

No major cardiovascular events (defined as severe hypotension, malignant arrhythmias, or cardiac arrest) were reported during anesthesia induction and maintenance prior to cardiopulmonary bypass (CPB). The mean CPB duration was 183.3 ± 55.6 min. The mean donor heart cold ischemia time was 249.5 ± 101.1 min. The mean postoperative mechanical ventilation duration was 7.7 ± 9.5 days.

### 3.3 Postoperative complications, ECMO support, and survival outcomes

After transplantation, 11 patients (40.7%) required ECMO support. The mean duration of postoperative ECMO support was 5 ± 3 days.At the end of follow-up, 23 patients were alive and 4 died, resulting in an overall survival rate of 85.2% (23/27). The reported causes of death were low cardiac output syndrome (*n* = 1), septic shock (*n* = 1), rejection-related malignant arrhythmia (*n* = 1), and intracranial infection with multiple cerebral infarctions (*n* = 1). Among the 4 deceased patients, one had a history of ependymoma and had undergone surgery and multiple courses of chemotherapy; this death was considered primarily related to non-cardiac causes. Postoperative complications involving multiple organ systems were observed, including hypoxic-ischemic encephalopathy (4 cases), acute kidney injury requiring continuous renal replacement therapy (5 cases), and peripheral nerve injury (2 cases).

## 4 Discussion

**Figure 1:**
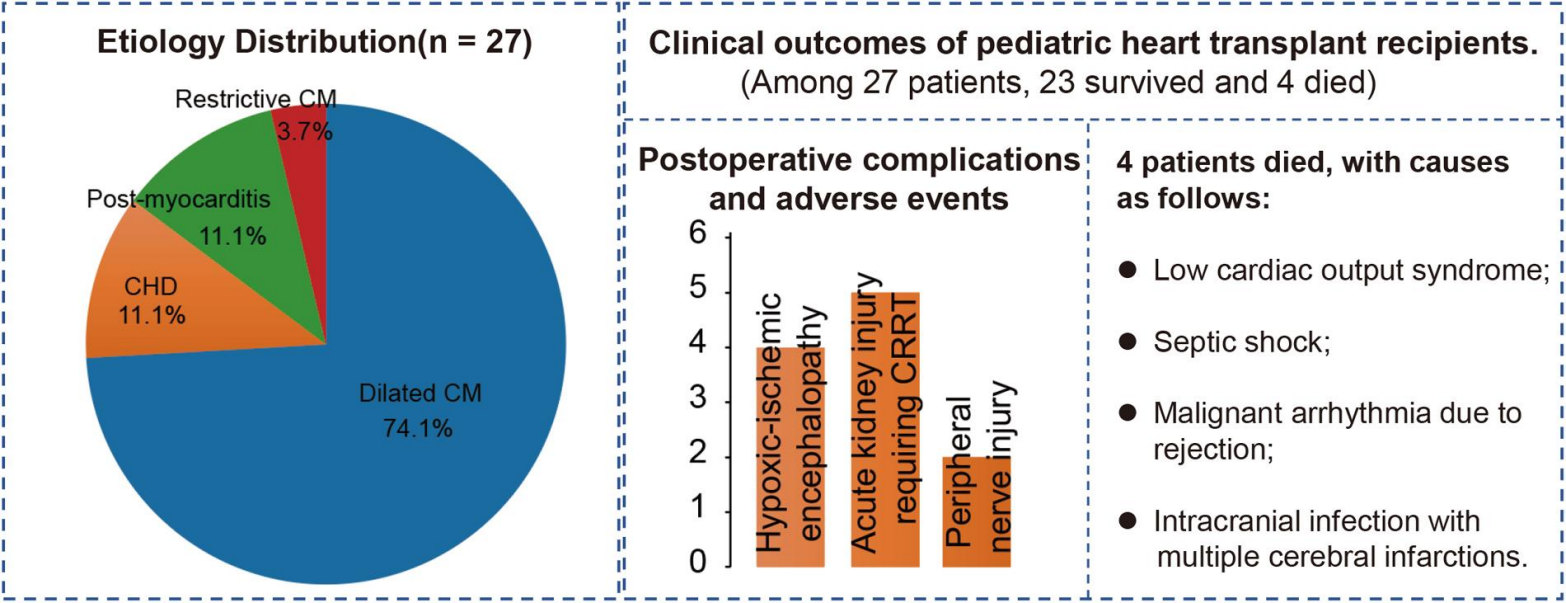
Etiologic distribution and major postoperative outcomes in pediatric heart transplant recipients. The figure summarizes the etiologic distribution and selected postoperative outcomes in 27 pediatric heart transplant recipi-ents. Dilated cardiomyopathy was the most common underlying diagnosis (74.1%). Four patients died during follow-up. Major complications included hypoxic-ischemic encephalopathy (*n* = 4), acute kidney injury requiring continuous renal replacement therapy (*n* = 5), and peripheral nerve injury (*n* = 2).

In this single-center descriptive study involving 27 pediatric heart transplant recipients, we documented perioperative anesthesia management strategies and short-term clinical outcomes for a cohort with a high proportion (55.6%) of patients receiving extracorporeal membrane oxygenation (ECMO) bridging therapy. No major cardiovascular events were reported during anesthesia induction and maintenance prior to cardiopulmonary bypass. The overall survival rate at the end of follow-up was 85.2% (23/27). Major complications observed included hypoxic-ischemic encephalopathy (4 cases), acute kidney injury requiring continuous renal replacement therapy (5 cases), and peripheral nerve injury (2 cases). Given that this study is descriptive, retrospective, single-center, and lacks a control group, these findings should be regarded as generating hypotheses rather than causal conclusions or recommendations for clinical practice (Figure 1).

### 4.1 Comparison with published literature

Since the first pediatric heart transplant was performed in 1967, the global annual surgical volume has steadily increased, reaching approximately 600 cases annually in recent years (11). Advances in surgical techniques, preoperative intensive care, anesthesia management, and multidisciplinary collaboration have contributed to improved short-and long-term survival rates among pediatric recipients. According to data from the International Society for Heart and Lung Transplantation (ISHLT), congenital heart disease is the primary indication for heart transplantation in children aged 1 year and younger, while cardiomyopathy predominates in children over 1 year of age (11). In this cohort, dilated cardiomyopathy was the most common primary diagnosis (74.1%), consistent with previous reports in children over 1 year of age. The proportion of congenital heart disease in this cohort was relatively low (11.1%), and all affected patients were older children (mean age: 13.3 ± 0.47 years). Possible explanations for this finding include the widespread use of prenatal screening, early intervention for complex malformations, prior history of palliative or corrective surgery, and the relative scarcity of suitable pediatric donors in China.

In this cohort, all patients presented with advanced heart failure at the time of transplantation, characterized by a median preoperative left ventricular ejection fraction of 28.5%, and all patients were classified as NYHA cardiac function class III or IV. These findings are consistent with other single-center pediatric heart transplant series reports.

### 4.2 Mechanical circulatory support as a transplantation bridge

For patients with end-stage heart failure whose hemodynamic status cannot be stabilized with monotherapy, mechanical circulatory support can serve as a bridging measure prior to transplantation. Recent international reports indicate that the proportion of pediatric patients undergoing mechanical support bridging transplantation increased from less than 20% between 2005 and 2008 to 43.2% (12). The selection of ventricular assist devices depends on the support strategy, anticipated duration of support, patient body size, and cardiac anatomy. However, due to physical constraints, the use of VADs remains limited in infants and low-weight children, with only a few devices (e.g., the Berlin Heart EXCOR) being suitable for this population (13). In contrast, ECMO remains the primary short-term bridging approach and postoperative circulatory support strategy for infants and young children undergoing heart transplantation.

In this cohort, 15 out of 27 patients (55.6%) received preoperative ECMO support. This proportion is higher than that reported in some international series. Patients receiving preoperative ECMO support exhibited a lower mean preoperative left ventricular ejection fraction compared to those who did not (29.07 ± 14.88% vs. 35.83 ± 12.87%). This difference may reflect more severe hemodynamic instability and overall disease severity in patients requiring preoperative ECMO support. Prolonged waiting times and donor scarcity may also contribute. However, since this study is descriptive and lacks a control group, we cannot determine whether these factors independently influenced the outcomes.

### 4.3 Anesthesia induction and intraoperative management

In children who have undergone heart transplantation, myocardial catecholamine depletion and downregulation of *β*-adrenergic receptors may lead to preoperative circulatory decompensation and reduced tolerance to anesthetic agents (14). In this cohort, no major cardiovascular events (including severe hypotension, malignant arrhythmias, or cardiac arrest) were reported during anesthesia induction and maintenance prior to cardiopulmonary bypass. This finding supports the clinical feasibility of a low-dose, slow, stepwise induction strategy in this high-risk population. Previous studies have demonstrated that etomidate, opioids, and ketamine may offer hemodynamic advantages over propofol and volatile anesthetics in patients with more severe cardiac conditions and higher American Society of Anesthesiologists (ASA) risk classifications (15, 16).

At our center, an induction protocol based on midazolam and medium-dose opioids was employed. No significant hemodynamic instability was reported with this approach. The selection of anesthetic agents in this cohort was individually tailored to maintain circulatory stability, with multimodal anesthesia and adjunctive positive inotropic support administered as needed.

### 4.4 Postoperative right heart failure and pulmonary hypertension

According to reports, the incidence of severe right heart failure post-heart transplantation ranges from approximately 15% to 43%, with elevated pulmonary capillary wedge pressure and increased mean pulmonary artery pressure identified as the primary risk factors (17). At our center, all patients underwent preoperative pulmonary artery pressure assessment, and older children additionally underwent right heart catheterization to evaluate pulmonary vascular resistance. For patients with elevated pulmonary artery pressure, pulmonary vasodilators such as treprostinil were administered preoperatively.

According to ISHLT recommendations, pulmonary vascular responsiveness testing should be performed when pulmonary arterial systolic pressure ≥50 mmHg, the transpulmonary gradient ≥15 mmHg, or pulmonary vascular resistance>3 Wood units. Patients with preoperative pulmonary arterial systolic pressure>60 mmHg or pulmonary vascular resistance>4–5 Wood units are generally considered unsuitable for pure heart transplantation (18). In this cohort, the intraoperative management strategy for patients with pulmonary hypertension included: using norepinephrine to maintain systemic hemodynamic stability, avoiding hypercapnia and acidosis, and employing ventilation strategies to limit airway pressure and right ventricular afterload. These practices are consistent with those described in other published reports.

### 4.5 Acute renal injury

Acute kidney injury (AKI) is relatively common following pediatric heart transplantation, with reported incidence rates ranging from 63% to 73% (19, 20). Postoperative AKI may result from pre-existing renal insufficiency associated with acute or chronic cardiorenal syndrome, intraoperative hemodynamic instability, or perioperative critical conditions including cardiogenic or septic shock (19). MacDonald et al. identified independent risk factors for postoperative AKI in 66 pediatric heart transplant recipients, including preoperative positive inotropic support, preoperative mechanical ventilation, lower estimated creatinine clearance, and tacrolimus levels>15 μg/L on day 3 postoperatively (22). In a retrospective cohort study involving 177 patients, Lipman et al. found that prolonged extracorporeal circulation time and higher body weight at transplantation were associated with an increased risk of severe AKI requiring dialysis (21).

In this cohort, 5 out of 27 patients (14.8%) required continuous renal replacement therapy due to severe perioperative AKI. Due to the small sample size and descriptive design, we were unable to identify independent risk factors for AKI in this cohort.

### 4.6 Limitations

The findings of this study should be interpreted within the context of its design limitations. First, this is a single-center retrospective descriptive study involving only 27 patients, resulting in a small sample size. This limitation reduced statistical power, preventing us from conducting multivariate analyses or identifying independent predictors of outcomes, and restricted the generalizability of the results to other centers or populations. Second, no control group was established. Consequently, we cannot conclude that any specific anesthesia strategy led to better or worse outcomes; we can only describe what was performed and observed. Third, although our perioperative management was consistent in principle, we did not adhere strictly to a written protocol, and certain practices may have undergone minor variations during the 3.5-year study period. Fourth, our follow-up period was short, focusing on in-hospital and early post-discharge outcomes; we lack data on long-term graft function, chronic rejection, or neurodevelopmental outcomes. Fifth, more than half of the patients (55.6%) required ECMO support prior to transplantation, indicating that our cohort represents a highly severe subgroup of pediatric heart transplant recipients; our findings may not be applicable to low-risk patients. Finally, as with all retrospective studies, we cannot completely exclude selection bias or information bias. Given.Given these limitations, our study should be regarded as a source of hypothetical observations rather than definitive guidance for clinical practice.

## 5 Conclusion

In this single-center descriptive study involving 27 pediatric heart transplant recipients, staged low-dose induction, comprehensive monitoring, and multidisciplinary perioperative management were observed in a cohort comprising multiple critically ill patients and ECMO bridge patients. No major cardiovascular events occurred during anesthesia induction. The overall survival rate at the end of follow-up was 85.2%. Pulmonary hypertension, primary graft dysfunction, and multi-organ complications were relatively common in this cohort. Given that the study was descriptive, retrospective, single-center, and lacked a control group, causal conclusions regarding specific anesthesia strategies cannot be drawn. These findings should be regarded as observational data generating hypotheses. Larger-scale, preferably prospective, multicenter studies are required to validate and expand these findings.

## Data Availability

The raw data supporting the conclusions of this article will be made available by the authors, without undue reservation.

## References

[1] Barnard CN. The operation. A human cardiac transplant: an interim report of a successful operation performed at Groote Schuur Hospital, Cape Town. S Afr Med J. (1967) 41(48):1271–4. 4170370

[2] Dipchand AI Webber SA. Pediatric heart transplantation: looking forward after five decades of learning. Pediatr Transplant. (2024) 28:e14675. 10.1111/petr.14675 38062996

[3] Huang JF. Report on The Development of Organ Donation and Transplantation in China (2024). Beijing: Tsinghua University Press (2025).

[4] Zhou C Wang GH Wang YX. Chinese operating specifications for pediatric heart transplantation. Chin J Transplant. (Electronic Edition). (2020) 14(3):136–42.

[5] Hsu DT Lamour JM. Changing indications for pediatric heart transplantation: complex congenital heart disease. Circulation. (2015) 131:91–9. 10.1161/CIRCULATIONAHA.114.001377 25561474

[6] Demirel A Özgünay ŞE Eminoğlu Ş Balkaya AN Onur T Kılıçarslan N. Ultrasonographic evaluation of gastric content and volume in pediatric patients undergoing elective surgery: a prospective observational study. Children (Basel). (2023) 10:1432. 10.3390/children10091432 37761393 PMC10529717

[7] Rivera Jiménez KE Mamani Ticona YM Gutierrez-Chavez G Astudillo CO Calle E Heredia GAT. Heparin resistance in cardiac surgery with cardiopulmonary bypass: mechanisms, clinical implications, and evidence-based management. Medicina (Kaunas). (2025) 61:2088. 10.3390/medicina61122088 41470090 PMC12734821

[8] Dammeyer KL Motonaga KS Chubb H Profita EL Hollander SA. Early post-operative versus late arrhythmias after heart transplant at a pediatric center: incidence, management, and outcomes. Pediatr Transplant. (2025) 29:e70094. 10.1111/petr.70094 40464372

[9] Gautam N Deniwar A Hubbard R Pawelek O Griffin E Edmonds K. Implementation of a standardized neonatal cardiac surgery protocol improves postoperative outcomes. J Am Coll Cardiol. (2021) 77(18 Suppl 1):492. 10.1016/S0735-1097(21)01851-9

[10] Seifert HA Jobes DR Ten Have T Kimmel SE Montenegro LM Steven JM. Adverse events after protamine administration following cardiopulmonary bypass in infants and children. Anesth Analg. (2003) 97:383–9. 10.1213/01.ANE.0000072545.13681.FA 12873922

[11] Singh TP Cherikh WS Hsich E Chambers DC Harhay MO Hayes D Jr. The international thoracic organ transplant registry of the international society for heart and lung transplantation: twenty-fourth pediatric heart transplantation report—2021; focus on recipient characteristics. J Heart Lung Transplant. (2021) 40:1050–9. 10.1016/j.healun.2021.07.022 34420853 PMC10281816

[12] Singh TP Hsich E Cherikh WS Perch M Hayes D Lewis A. The international thoracic organ transplant registry of the international society for heart and lung transplantation: 2025 annual report of heart and lung transplantation. J Heart Lung Transplant. (2025) 44:1857–73. 10.1016/j.healun.2025.04.014 40300677

[13] Rohde S Antonides CFJ Dalinghaus M Muslem R Bogers AJJC. Clinical outcomes of paediatric patients supported by the Berlin heart EXCOR: a systematic review. Eur J Cardiothorac Surg. (2019) 56:830–9. 10.1093/ejcts/ezz092 30932146

[14] Rosenthal DN Hammer GB. Cardiomyopathy and heart failure in children: anesthetic implications. Paediatr Anaesth. (2011) 21:577–84. 10.1111/j.1460-9592.2011.03561.x 21481080

[15] Yuki K Lee S Staffa SJ DiNardo JA. Induction techniques for pediatric patients with congenital heart disease undergoing noncardiac procedures are influenced by cardiac functional status and residual lesion burden. J Clin Anesth. (2018) 50:14–7. 10.1016/j.jclinane.2018.06.022 29936283

[16] Sarkar M Laussen PC Zurakowski D Shukla A Kussman B Odegard KC. Hemodynamic responses to etomidate on induction of anesthesia in pediatric patients. Anesth Analg. (2005) 101:645–50. 10.1213/01.ane.0000166764.99863.b417 16115968

[17] Hoskote A Carter C Rees P Elliott M Burch M Brown K. Acute right ventricular failure after pediatric cardiac transplant: predictors and long-term outcome in current era of transplantation medicine. J Thorac Cardiovasc Surg. (2010) 139:146–53. 10.1016/j.jtcvs.2009.08.020 19910002

[18] Nesseler N Mansour A Cholley B Coutance G Bouglé A. Perioperative management of heart transplantation: a clinical review. Anesthesiology. (2023) 139:493–510. 10.1097/ALN.000000000000462719 37458995

[19] Patel M Heipertz A Joyce E Kellum JA Horvat C Squires JE. Acute kidney disease predicts chronic kidney disease in pediatric non-kidney solid organ transplant patients. Pediatr Transplant. (2022) 26:e14172. 10.1111/petr.14172 34668615 PMC9018890

[20] Leghrouz B Kaddourah A. Impact of acute kidney injury on critically ill children and neonates. Front Pediatr. (2021) 9:635631. 10.3389/fped.2021.635631 33981652 PMC8107239

[21] Lipman AR Lytrivi ID Fernandez HE Lynch AM Yu ME Stevens JS. Acute kidney injury requiring dialysis after pediatric heart transplant. Pediatr Transplant. (2024) 28:e14829. 10.1111/petr.14829 39036942 PMC11268797

[22] MacDonald C Norris C Alton GY Urschel S Joffe AR Morgan CJ. Acute kidney injury after heart transplant in young children: risk factors and outcomes. Pediatr Nephrol. (2016) 31:671–8. 10.1007/s00467-015-3252-x 26559064

